# Colorectal laterally spreading tumors exhibit a distinct carcinogenic eco-metabolic shift defined by multi-omics profiling

**DOI:** 10.3389/fmicb.2026.1887252

**Published:** 2026-08-10

**Authors:** Wenming Liu, Jie Liu, Yudai Chen, Guijun Lu, Jiaxin Shen, Xiaoling Zheng, Xujin Wei

**Affiliations:** 1 Endoscopic Center, The First Affiliated Hospital, Fujian Medical University, Fuzhou, Fujian, China; 2 Endoscopic Center, National Regional Medical Center, Binhai Campus of the First Affiliated Hospital, Fujian Medical University, Fuzhou, Fujian, China; 3 Digestive Endoscopy Center, Fuzhou University Affiliated Provincial Hospital, Fuzhou, Fujian, China

**Keywords:** colorectal tumorigenesis, gut microbiome, LST, microbial metabolism, Roseburia

## Abstract

**Background:**

Colorectal laterally spreading tumors (LSTs) are clinically important premalignant lesions with distinctive endoscopic morphology and malignant potential. Although microbiome and metabolome alterations have been reported in colorectal cancer and conventional adenomas, the microbial and metabolic features associated with LSTs remain insufficiently characterized.

**Objective:**

We investigated whether LSTs are associated with a coordinated carcinogenic eco-metabolic shift (CES) involving stool microbial, metagenomic functional, and circulating metabolic alterations, hereafter referred to a CES, a finding consistent with its high-risk premalignant biology.

**Methods:**

Building on our LST multi-omics cohort, we focused on stool metagenomic, serum metabolomic, and paired stool–serum datasets from 35 LST patients and 35 healthy controls. LST-associated microbial taxa, serum metabolites, and Kyoto Encyclopedia of Genes and Genomes (KEGG) functional features were organized into CES modules according to their biological direction and functional themes. Cross-layer Spearman’s analyses, functional pathway analyses, and sign-aligned CES scores were used to assess coordination across microbial, functional, and metabolic layers. Publicly available MetaGenoPolis stool metagenomic data were used as a reference cohort for comparisons across healthy controls, adenoma, and colorectal cancer (CRC) disease sequences.

**Results:**

LST patients showed a CES characterized by the depletion of protective anaerobe-associated taxa; enrichment of facultative/pathobiont-associated taxa; remodeling of dicarboxylate/TCA axis metabolism; alterations in amino acid and choline/glycerophospholipid metabolism; and enrichment of microbial functions related to carbohydrate uptake, central carbon metabolism, transport, and biofilm-associated adaptation. In paired stool–serum samples, four cross-layer associations were retained, including an exploratory inverse *Roseburia*–succinate relationship. In the MetaGenoPolis cohort, the matched microbial component of the CES showed a CRC-oriented pattern: CRC patients had higher microbial CES scores than healthy controls and adenoma patients, whereas the microbial CES scores of healthy controls and adenoma patients were not significantly different. Sign-aligned CES scores were higher in LST patients than in healthy controls across stool, serum, and paired multi-omics analyses; the paired integrated CES score showed a rank-biserial effect size of 0.952. A compact five-anchor CES representation, based on *Roseburia* depletion, succinate elevation, phosphotransferase system (PTS) elevation, 3-hydroxybutyric acid depletion, and *Escherichia* enrichment, preserved the main signal.

**Conclusion:**

LSTs are associated with a coordinated CES involving stool microbial, microbial functional, and serum metabolic alterations. These findings support the CES as a testable framework for understanding high-risk premalignant colorectal biology and warrant validation in larger cohorts with adenoma and serrated lesion comparators.

## Introduction

Laterally spreading tumors (LSTs) are a distinct endoscopic phenotype of colorectal premalignant lesions that spread laterally along the mucosal surface rather than forming a primarily protruded mass (Djinbachian et al., 2024). Their large size and non-polypoid morphology make them technically and biologically important in gastrointestinal endoscopy (Zhong et al., 2026; Bonniaud et al., 2022). Since a subset of LSTs show advanced histology or malignant potential, they are clinically relevant to colorectal cancer (CRC) prevention and are not fully represented by common polypoid adenoma models (Li et al., 2021). These features raise an important biological question: Do LSTs already carry microbial and metabolic alterations associated with high-risk colorectal tumorigenesis?

The gut microbiome has been repeatedly implicated in colorectal neoplasia, with proposed roles in inflammation, epithelial signaling, microbial genotoxicity, and metabolic remodeling (Hu et al., 2025; Li Q. et al., 2025). CRC metagenomic studies have identified recurrent microbial signatures across cohorts, including enrichment of taxa such as *Fusobacterium nucleatum* and other pathobiont-associated organisms (Zepeda-Rivera et al., 2024; Osswald et al., 2025). Experimental and human evidence further links microbial genotoxicity, biofilm-associated organization, and toxin-producing bacteria with colorectal tumorigenesis (Liu et al., 2026). Studies across the healthy–adenoma–CRC sequence suggest that microbial and functional shifts can emerge before the development of invasive cancer (Lin Y. et al., 2025), and multi-cohort analyses have shown that CRC-associated microbial signatures are generalizable across populations (Piccinno et al., 2025). However, a majority of colorectal microbiome studies are organized around healthy controls, conventional adenomas, and CRC. LSTs are underrepresented in this disease-sequence framework, and it remains unclear whether their microbial and metabolic features resemble those of conventional adenomas, CRC-oriented dysbiosis, or a distinct high-risk premalignant state.

Multi-omics analyses provide an opportunity to connect microbial ecology with host-facing metabolic consequences. Stage-resolved metagenomic and metabolomic analyses in colorectal neoplasia have shown that microbial and metabolic phenotypes can vary across disease progression (Sun et al., 2024). Microbial metabolites and host-facing metabolic readouts are currently widely viewed as essential for interpreting whether a microbial pattern is biologically meaningful in colorectal neoplasia (Wong and Yu, 2023; Almonte et al., 2026). Short-chain fatty acid and butyrate-related biology remains important for mucosal homeostasis; however, recent evidence also highlights that metabolite interpretation should be axis-specific, disease stage-aware, and integrated with microbial function rather than reduced to a single protective-versus-harmful label (Alvandi et al., 2022). For LSTs, paired stool metagenomics and serum metabolomics are therefore useful because they can test whether anaerobe depletion, facultative/pathobiont-associated enrichment, microbial functional programs, and circulating metabolic remodeling form one coherent carcinogenic eco-metabolic shift (CES).

In this study, we extend our LST stool metagenomic and serum metabolomic research by asking whether the microbial, functional, and metabolic signals form coherent high-risk premalignant biology (Lin H. et al., 2025). LST-associated microbial, functional, and serum metabolomic features were organized within a carcinogenic eco-metabolic shift (CES) framework. Publicly available MetaGenoPolis stool metagenomic data were then used as a reference disease-sequence cohort for the microbial component that could be matched across datasets (Plaza Oñate et al., 2019). We hypothesized that LSTs are associated with a CES involving protective anaerobe depletion, facultative/pathobiont-associated enrichment, serum metabolic remodeling, and quantifiable sample-level displacement toward high-risk colorectal tumorigenesis ecology.

## Materials and methods

### Study design and data sources

This study utilized fecal metagenomic and serum metabolomic data from our previously reported LST cohort (Lin H. et al., 2025). The full cohort comprised 63 LST patients and 85 healthy controls. In that study, LST patients and healthy controls were matched at a ratio of 1:1 using propensity score matching, with age, sex, and BMI considered as covariates and a caliper value of 0.02, yielding a final cohort of 35 LST patients and 35 healthy controls. Post-matching baseline characteristics were reported in the prior publication (Lin H. et al., 2025). Detailed procedures for participant enrollment, sample collection, DNA extraction, metagenomic sequencing, and untargeted metabolomic profiling have been described previously. Raw sequencing data have been deposited in the NCBI BioProject database under accession number PRJNA1212478.

### Feature organization and paired cross-layer analysis

LST-associated stool microbial taxa, serum metabolites, and stool KEGG functional features were organized according to biological direction and CES module. LST-enriched and LST-depleted features were retained as separate biological signals during descriptive analyses. For CES scoring, feature directions were aligned so that higher values represented a stronger expression of the LST-associated CES. Functional modules included protective anaerobe depletion, facultative/pathobiont-associated enrichment, dicarboxylate/TCA metabolism, amino acid remodeling, choline/glycerophospholipid metabolism, carbohydrate transport, biofilm-associated adaptation, and central carbon metabolism.

Cross-layer coordination was assessed in the paired stool–serum subset. Prespecified microbial taxa, serum metabolites, and KEGG functional features were assembled into a subject-level feature matrix. Spearman’s correlations were tested across cross-layer feature pairs. Displayed associations were selected using predefined statistical and biological criteria and were interpreted as evidence of coordination rather than causal interactions.

### Public microbial disease-sequence comparison

LST-associated microbial features were matched to publicly available MetaGenoPolis microbial features. A broad microbial CES score and a taxa-only CES sensitivity score were calculated across healthy control, adenoma, and CRC groups. Additional mapping-restricted sensitivity analyses were conducted to evaluate whether the observed pattern depended on broad feature matching or functional proxy features. Disease-sequence comparisons were performed using the public Recherche Data Gouv/MetaGenoPolis dataset.^1^ This dataset aggregates 2,340 human stool shotgun metagenomes from 15 public colorectal cancer-related cohorts across 10 countries and provides manually curated metadata, METEOR taxonomic profiles, and METEOR functional module profiles. As serum metabolomic data were unavailable and public cohorts were heterogeneous, this analysis was interpreted as providing a stool microbial disease-sequence context rather than validating the full multi-omics CES framework.

Functional pathway analysis.

Internal stool metagenomic KEGG features were evaluated to identify functional programs compatible with the LST-associated CES. Main functional candidates were selected from KEGG rows related to carbohydrate uptake, transport, central carbon metabolism, and biofilm-associated adaptation. Carbohydrate-Active EnZymes (CAZy) families were analyzed as supportive evidence of carbohydrate-active enzyme potential. Public species-stratified pathway-contributor evidence from FengQ_2015 was retained as an exploratory supplementary context only and was not used as proof of species-resolved functional replacement. The source cohort was derived from the colorectal adenoma–carcinoma sequence study by Feng et al. and contained stool shotgun metagenomic samples from the healthy control, adenoma, and CRC groups (Feng et al., 2015). We retrieved the HUMAnN3-style ‘pathway_abundance’ resource and used species-stratified pathway rows in the ‘pathway|taxon’ format.

CES scoring and five-anchor CES sensitivity analysis.

A sign-aligned CES score was calculated using primary stool taxa, serum metabolites, and stool KEGG functional features. For each feature, values were aligned so that higher scores reflected stronger expression of the LST-associated CES. Scores were summarized separately for stool, serum, and paired integrated multi-omics samples. This score was designed as a quantitative summary measure rather than as a diagnostic test.

To improve biological interpretability, a compact five-anchor CES score was constructed from *Roseburia* depletion, succinate elevation, PTS elevation, 3-hydroxybutyric acid depletion, and *Escherichia* enrichment. These features represented protective anaerobe depletion, dicarboxylate/TCA axis remodeling, carbohydrate uptake, ketone/butyrate-related metabolism, and facultative/pathobiont-associated enrichment. This analysis was used as a sensitivity and interpretation layer to test whether the broader CES could be summarized by a small set of biologically readable features.

### Statistical analysis

Rank-biserial effect sizes, non-parametric group comparisons, Spearman’s correlations, and Benjamini–Hochberg false discovery rate (FDR) adjustment were used according to the analysis layer. Public disease-sequence comparisons were interpreted using pairwise FDR-adjusted contrasts among the healthy control, adenoma, and CRC groups. CES scores were used as sign-aligned summaries of a predefined biological pattern, without prespecifying a clinical cutoff. The analyses used processed local source tables and documented scripts. Python analyses were performed using Python 3.12.13 with pandas 3.0.1 and numpy 2.3.5; final figures were generated using R 4.6.0.

## Results

### LST multi-omics data define a carcinogenic eco-metabolic shift

In this study, we used the LST multi-omics dataset to ask a question distinct from feature discovery: Whether LST-associated microbial, functional, and circulating metabolic signals form a coordinated carcinogenic eco-metabolic shift (CES) that can be quantified and positioned within colorectal disease progression.

**Figure 1:**
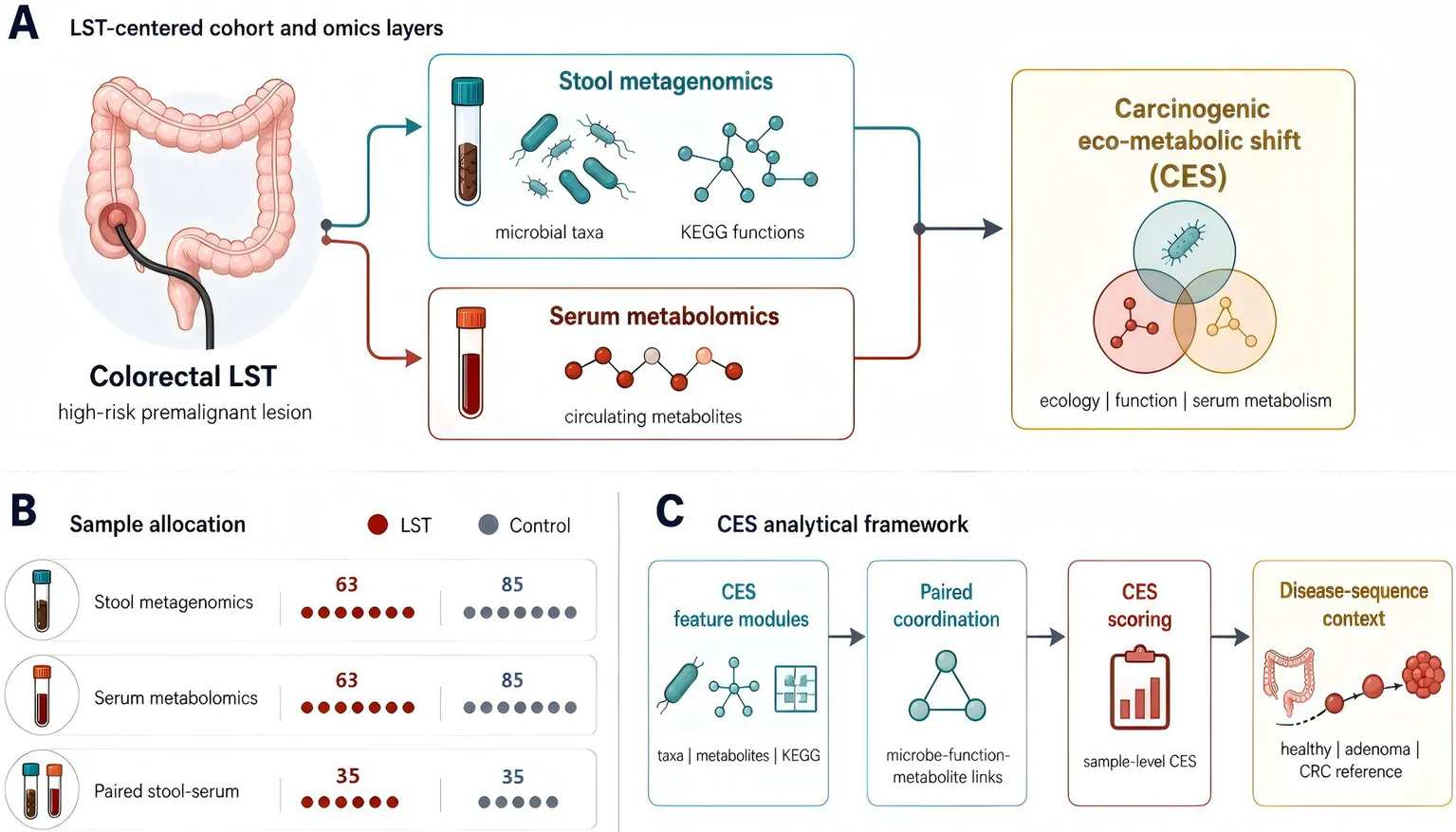
LST multi-omics study design and analytical framework. **(A)** Clinical LST–control cohort and paired biospecimen collection for stool metagenomics and serum metabolomics. **(B)** Analysis-ready LST and normal-control sample sets for stool metagenomics, serum metabolomics, and paired stool–serum analysis. **(C)** Analytical framework linking LST–control feature definition, paired multi-omics coordination, sample-level scoring, and public stool microbial disease-sequence comparison.

The CES framework was defined as a direction-aware organization of four biological components: loss of protective anaerobic ecology, expansion of facultative/pathobiont-linked features, remodeling of circulating metabolic axes, and enrichment of microbial functions related to nutrient uptake and adaptive stress programs (Figure 1A). The internal stool metagenomic and serum metabolomic datasets formed the evidentiary core of the analysis, with a strict matched stool–serum subset used for cross-layer coordination testing (Figure 1B). Public healthy–adenoma–colorectal cancer cohorts were then used for a narrower purpose: to place the mappable stool microbial component of the LST-derived CES along an external disease sequence rather than to replace the internal multi-omics evidence (Figure 1C).

### Microbial, functional, and serum metabolic features converge on CES modules

**Figure 2:**
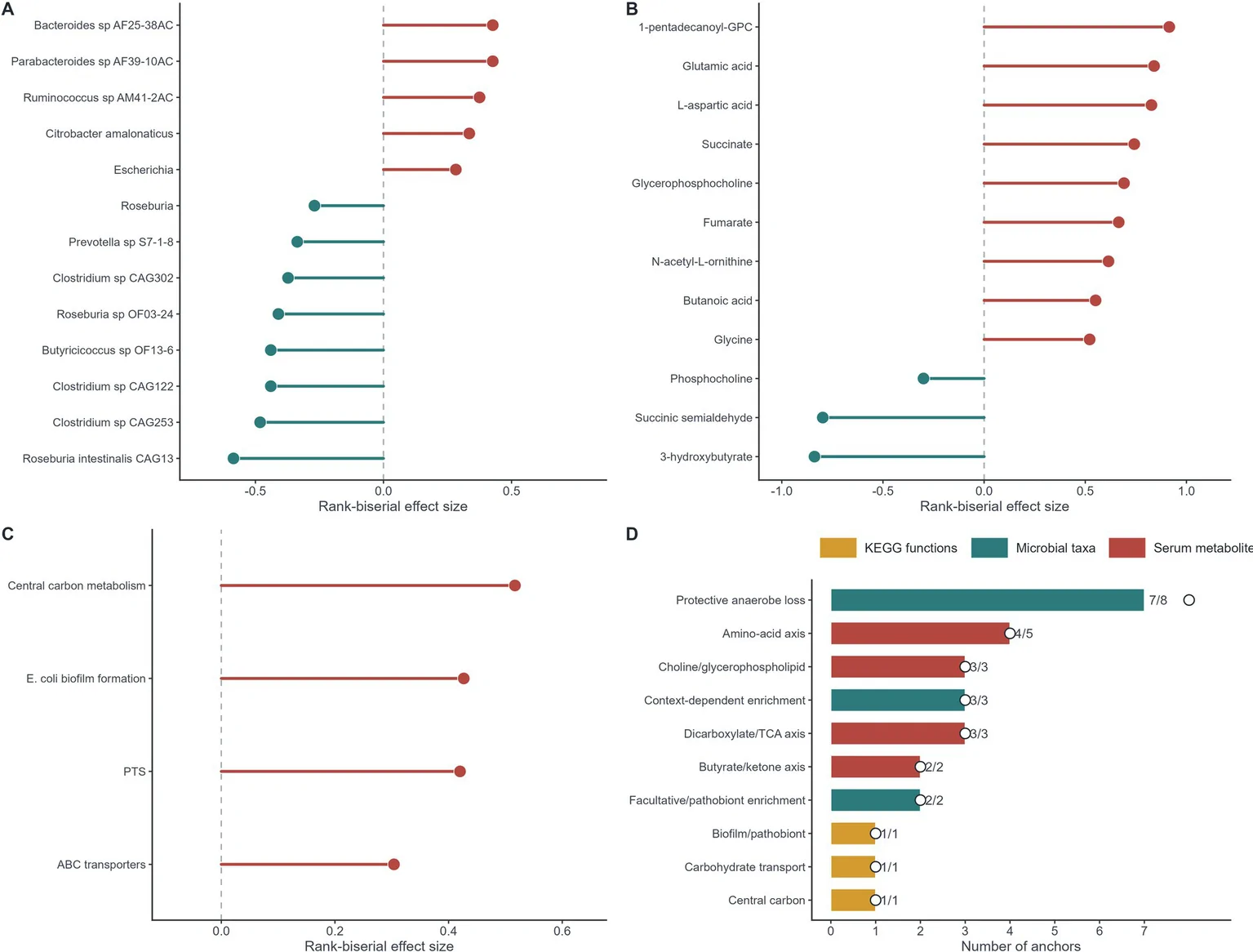
Microbial, functional, and serum metabolic features define the LST-associated CES. **(A)** Microbial features organized by ecological direction. **(B)** Serum metabolic features organized by the biological axis. **(C)** KEGG functional features related to carbohydrate uptake, transporter activity, biofilm-associated adaptation, and central carbon programs. **(D)** Module-level convergence across microbial, metabolic, and functional features.

We first reorganized the LST-associated features by biological direction and module rather than by data type alone. The microbial component was dominated by a shift away from anaerobic, fermentative gut ecology and toward a more facultative/pathobiont-permissive configuration. Protective anaerobe-linked anchors, including *Roseburia*-related features, *Butyricicoccus* sp. OF13-6, and several *Clostridium* CAG features, contributed to the depletion arm of the CES model, whereas *Escherichia* and *Citrobacter amalonaticus* contributed to its facultative/pathobiont arm (Figure 2A).

The serum metabolomic anchors provided a host-side counterpart to this microbial ecology. LST-associated signals mapped mainly to succinate/TCA–dicarboxylate metabolism, amino acid metabolism, choline/glycerophospholipid remodeling, and a butyrate/ketone-related axis marked by lower 3-hydroxybutyric acid levels (Figure 2B). These modules were treated as coordinated metabolic axes rather than as an expanded biomarker list.

Microbial function completed the CES definition. LST-enriched KEGG anchors were concentrated around carbohydrate uptake, transporter activity, biofilm/pathobiont adaptation, and central carbon programs (Figure 2C). When all internal anchors were summarized by module, the highest support converged on protective anaerobe depletion, facultative/pathobiont enrichment, dicarboxylate/TCA metabolism, amino acid remodeling, choline/glycerophospholipid metabolism, carbohydrate transport, biofilm adaptation, and central carbon metabolism (Figure 2D). Thus, the CES signature is best interpreted as a coordinated ecological and metabolic displacement rather than as a catalogue of isolated case–control differences.

### Matched multi-omics analysis links the microbial and metabolic arms of the CES

**Figure 3:**
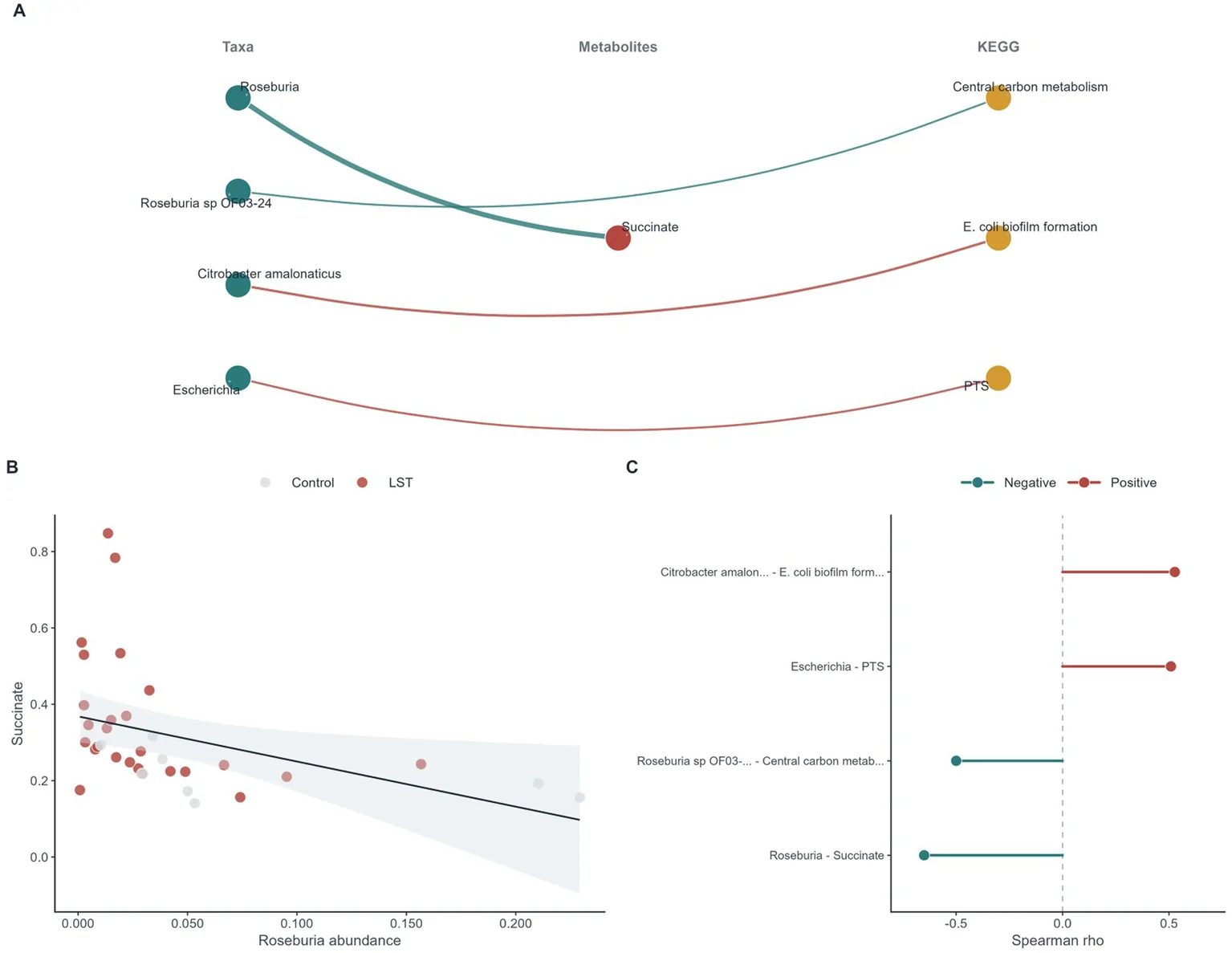
Paired multi-omics coordination among LST-associated features. **(A)** Focused cross-layer network showing displayed associations among microbial taxa, serum metabolites, and KEGG functions in paired samples. **(B)** *Roseburia*–succinate relationship. **(C)** *Spearman rho* summary for the four direction-compatible displayed associations. The network is supportive coordination evidence rather than a mechanistic causal map.

Having defined the CES modules, we next asked whether these layers aligned within the same individuals. In the strictly matched stool–serum subset, 31 CES nodes spanning microbial taxa, serum metabolites, and KEGG functional features were retained for prespecified cross-layer testing (Figure 3A). The displayed network was intentionally narrow: It was designed to highlight CES-compatible links among the main biological axes rather than to reproduce a broad correlation matrix.

In total, four cross-layer edges met the display criteria. The strongest taxa–metabolite link connected lower *Roseburia* with higher succinate levels (rho = −0.650, FDR = 0.062; Figure 3B), joining two central components of the model: anaerobe depletion and dicarboxylate/TCA axis displacement. As this edge did not pass the conventional FDR of < 0.05 threshold, it was interpreted as exploratory cross-layer support. The remaining display edges connected *Citrobacter amalonaticus* with *Escherichia coli* biofilm formation, *Escherichia* with PTS, and *Roseburia* sp. OF03-24 with central carbon metabolism in cancer (Figure 3C). These relationships support the interpretation that CES components are not merely parallel feature-level contrasts; rather, key microbial, functional, and serum metabolic axes aligned within the matched samples. The next question was as follows: Once a coordinated LST-associated state is observed internally, where does its stool microbial component sit along the broader healthy–adenoma–CRC sequence?

### Public disease-sequence comparison places the microbial CES component in a CRC-oriented context

**Figure 4:**
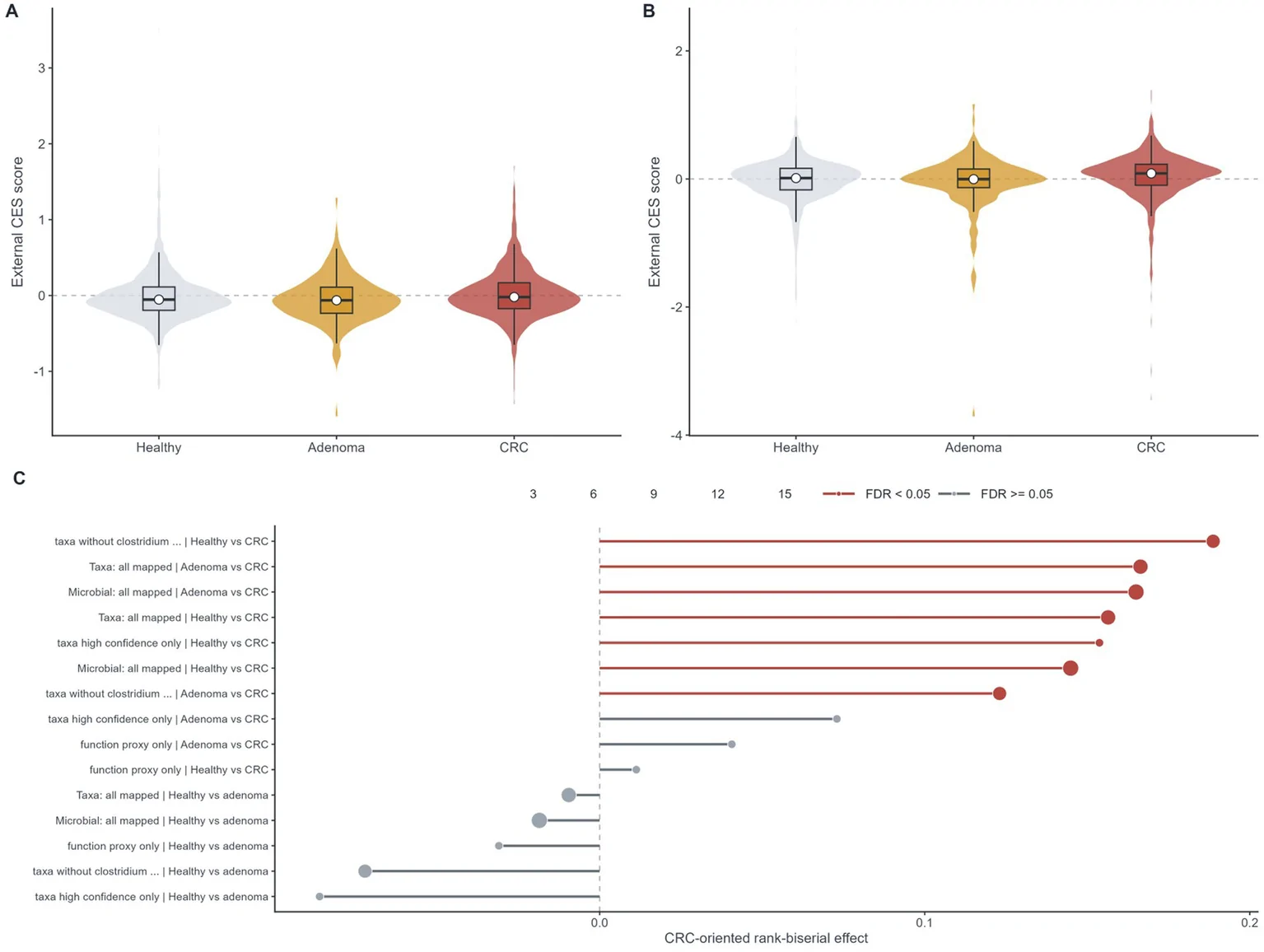
Public disease-sequence comparison of the stool microbial component. **(A)** Broad microbial composite score distributions across healthy controls, conventional adenoma patients, and CRC patients in MetaGenoPolis. **(B)** Taxa-only sensitivity score distributions. **(C)** Mapping-restricted sensitivity contrasts summarizing CRC-oriented score shifts. This analysis provides a disease-sequence context and should not be interpreted as validation of the paired stool–serum multi-omics pattern.

We projected the mappable stool microbial component of the internal CES signature into an external MetaGenoPolis healthy–adenoma–colorectal cancer cohort comprising 1,056 healthy controls, 185 patients with adenoma, and 897 patients with colorectal cancer (Figure 4A).

The broader external microbial CES score was most clearly shifted in colorectal cancer. Patients with CRC had higher microbial CES scores than healthy controls (FDR = 0.00785) and those with adenoma (FDR = 0.0254), whereas healthy controls and patients with conventional adenoma were not significantly separated (FDR = 0.5777; Figure 4B). A taxa-only sensitivity score sharpened the same pattern: CRC patients had higher scores than healthy controls (FDR = 2.20 × 10^−8^) and adenoma patients (FDR = 0.00162), while the healthy–versus–adenoma comparison remained weak (FDR = 0.8361; Figure 4C).

These results position the LST-derived microbial CES component in a CRC-oriented ecological context. It provides a disease-sequence context for a state first defined in internal LST multi-omics data: The broader microbial CES pattern is more evident at the CRC end of the public sequence than in conventional adenoma.

### LST metagenomes are enriched for functional programs compatible with CES ecology

**Figure 5:**
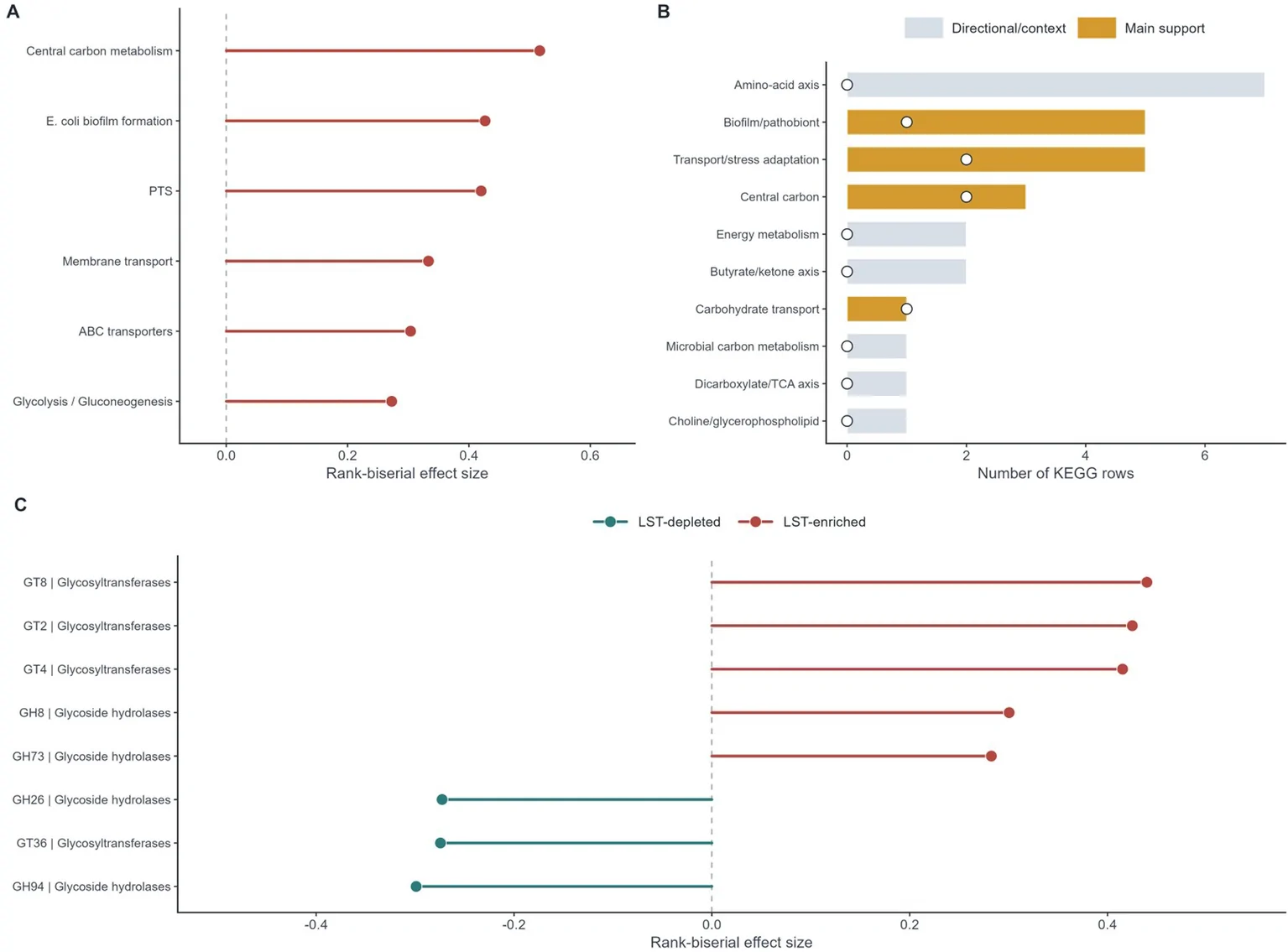
Functional ecology supporting the LST-associated CES. **(A)** Main KEGG candidates enriched in LST. **(B)** Functional theme support summarizing KEGG evidence. **(C)** Compact CAZy support shown as a supplementary functional context for altered carbohydrate-active enzyme potential.

We then returned to the internal stool metagenomes to determine which functional behaviors support the CES model. Among 460 tested KEGG rows, 28 belonged to CES-relevant functional themes, and 6 were retained as main-panel candidates (Figure 5A). The strongest candidates were enriched in LST and clustered around nutrient acquisition, central carbon remodeling, and adaptive microbial behavior.

The KEGG pathway annotated as central carbon metabolism in cancer showed the largest effect among the main functional candidates (rank-biserial effect size = 0.517, theme FDR = 6.31 × 10–4). In this metagenomic context, the pathway name was interpreted as a microbial functional annotation related to central carbon activity rather than as direct evidence of tumor cell metabolism. PTS (rank-biserial effect size = 0.420, theme FDR = 0.00260) and *E. coli* biofilm formation (rank-biserial effect size = 0.427, theme FDR = 0.0111) provided additional support for carbohydrate uptake and pathobiont adaptation axes (Figure 5B). Membrane transport, ABC transporters, and glycolysis/gluconeogenesis extended the same functional pattern, remaining directionally enriched in LST and supporting a metagenomic profile compatible with transporter activity and carbohydrate-processing capacity (Figure 5C).

CAZy families were used as supplementary functional support rather than as a primary claim because they provide a broader view of carbohydrate-active potential (Figure 5C). Taken together, the functional analysis gives biological meaning to the microbial CES component: The LST-associated community is not only taxonomically shifted but is also functionally oriented toward nutrient uptake, central carbon activity, transport, and biofilm/pathobiont adaptation.

### CES scoring summarizes the LST-associated multi-omics state at the sample level

**Figure 6:**
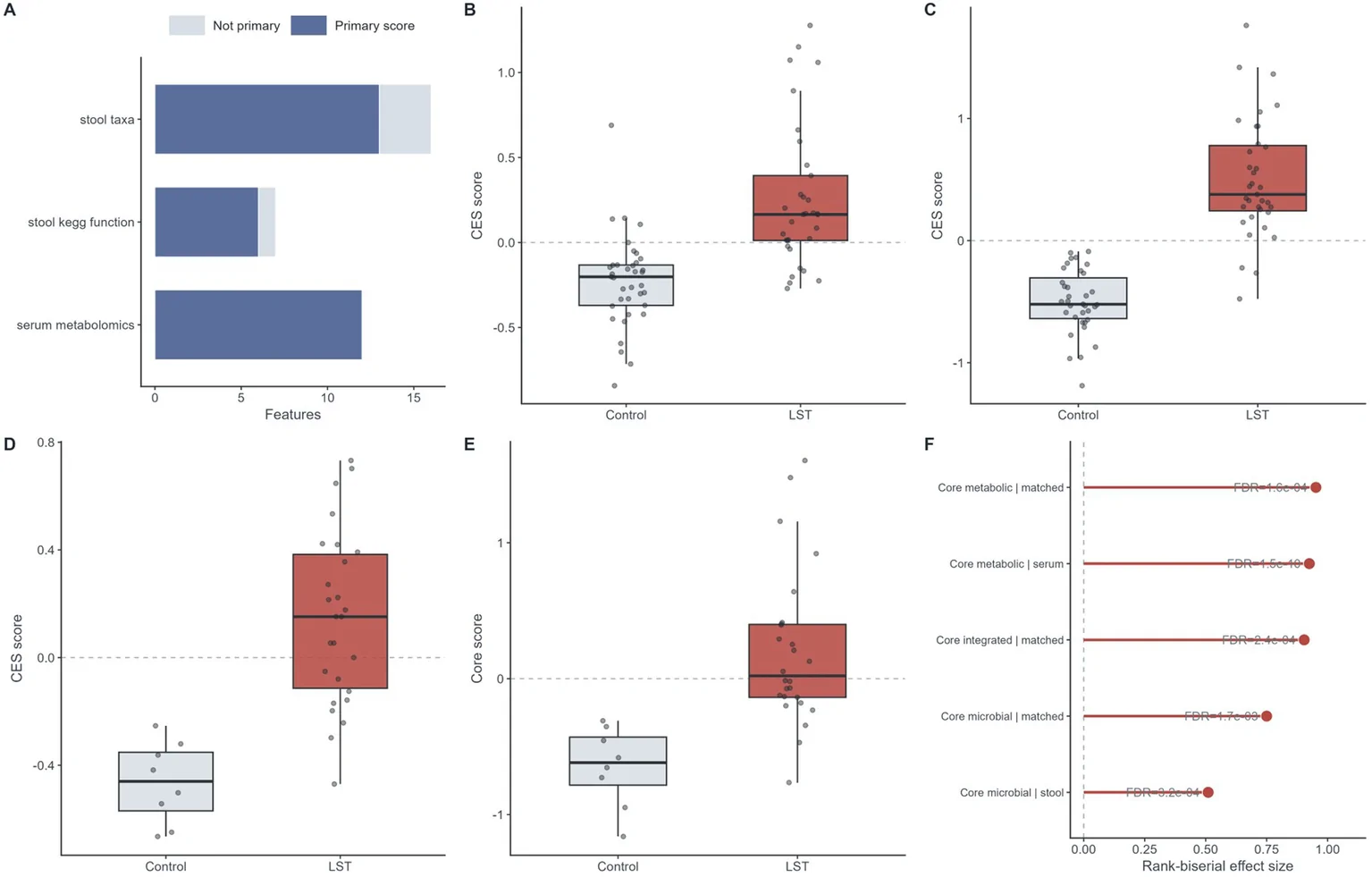
CES scoring and five-anchor sensitivity analysis. **(A)** Primary CES feature catalogue across stool taxa, stool KEGG functions, and serum metabolomics. **(B)** Combined stool CES score. **(C)** Serum metabolite CES score. **(D)** Integrated CES score in paired samples. **(E)** Integrated five-anchor CES score in paired samples. **(F)** Stool microbial, serum metabolic, and integrated five-anchor comparisons showing preservation of the broader CES signal.

After defining the CES at the feature, module, network, external-positioning, and functional levels, we asked whether the same state could be measured within individual samples. We constructed a direction-aware CES score in which LST-enriched anchors contributed positively and LST-depleted protective anchors contributed following sign reversal (Figure 6A). The score was intended as a sample-level summary of the predefined CES state, not as a diagnostic classifier.

Across stool metagenomic layers, LST patients had consistently higher CES scores than healthy controls. The taxa CES score was higher in LST patients (median 0.127 vs. −0.102; rank-biserial effect size = 0.713; FDR = 8.24 × 10^−7^), as was the KEGG function CES score (median 0.169 vs. −0.269; rank-biserial effect size = 0.482; FDR = 7.23 × 10^−4^). The combined stool CES score preserved this separation (median 0.165 vs. −0.202; rank-biserial effect size = 0.740; FDR = 4.48 × 10^−7^; Figure 6B).

The serum metabolite CES score showed the strongest layer-specific separation (median 0.378 vs. −0.521; rank-biserial effect size = 0.953; FDR = 5.99 × 10^−11^; Figure 6C). In the strictly matched multi-omics subset, the integrated CES score was also higher in LST patients (median 0.152 vs. −0.460; rank-biserial effect size = 0.952; FDR = 1.02 × 10^−4^; Figure 6D). The matched KEGG sub-score was directionally consistent but weaker than the taxa and serum components; therefore, the integrated matched signal should be interpreted mainly as coordinated microbial and serum metabolic displacement with functional support.

These scoring results indicate that the CES is visible as a direction-aware internal feature signature, supported by matched cross-layer coordination, positioned within a CRC-oriented microbial context using public data, reinforced by functional ecology, and measurable as an individual-sample eco-metabolic displacement.

A five-anchor CES representation preserves the main biological signal.

Finally, we condensed the broader CES into five anchors that capture its main biological direction: *Roseburia* depletion, succinate elevation, PTS elevation, 3-hydroxybutyric acid depletion, and *Escherichia* enrichment (Figure 6E). This five-anchor representation summarizes a transition from anaerobic fermentative ecology toward a state marked by succinate accumulation, microbial carbohydrate uptake capacity, reduced ketone/butyrate-related metabolism, and facultative/pathobiont expansion.

The five-anchor representation retained the internal signal of the broader CES score. The stool core microbial score was higher in LST patients than in healthy controls (rank-biserial effect size = 0.510; FDR = 0.000316), and the serum core metabolic score showed stronger separation (rank-biserial effect size = 0.925; FDR = 1.48 × 10–10). In matched samples, the integrated five-anchor score remained elevated in samples from LST patients (rank-biserial effect size = 0.904; FDR = 0.000245; Figure 6F).

This compact model provides a readable endpoint, making the core biology of the LST-associated CES interpretable: loss of *Roseburia*-linked anaerobic ecology, higher succinate, higher microbial carbohydrate-uptake potential, lower 3-hydroxybutyrate, and higher *Escherichia*-linked facultative ecology.

## Discussion

This study defines a carcinogenic eco-metabolic shift in colorectal LST using stool metagenomic, microbial functional, and serum metabolomic features. The key contribution is not the discovery of an isolated microbial marker or metabolite; rather, the results organize LST-associated features into a coordinated pattern: the depletion of protective anaerobe-associated taxa, enrichment of facultative/pathobiont-associated taxa, microbial functions related to nutrient uptake and adaptive behavior, and serum metabolic remodeling along the dicarboxylate/TCA, amino acid, choline/glycerophospholipid, and ketone-related axes. This interpretation is consistent with the recent direction of the CRC microbiome field, which increasingly treats microbial composition, strain-level behavior, functional capacity, and host metabolic context as interdependent components of tumor-associated ecology (White and Sears, 2024; Ji et al., 2026).

The LST-centered framing is clinically important. Recent endoscopic guidance and practice discussions emphasize that large non-pedunculated colorectal lesions require lesion-specific optical assessment and resection planning because morphology, size, location, and invasion risk influence management (Ferlitsch et al., 2024; Pimentel-Nunes et al., 2022). Our data add a complementary biological layer: LST may also be accompanied by a stool–serum CES that is not adequately represented by a conventional marker catalogue. This does not mean that the CES is specific to LST relative to every other premalignant lesion. Rather, it means that LST provides a clinically meaningful setting in which high-risk endoscopic morphology can be examined together with microbial ecology and systemic metabolic displacement.

The microbial arm of the CES is characterized by two linked directions. First, *Roseburia*-related and other anaerobe-associated features were depleted, supporting a shift away from a fermentative gut ecology (Dong et al., 2024). Recent research on short-chain fatty acid biology continues to support the importance of microbial fermentation products for host physiology; however, it also cautions that metabolite interpretation depends on disease context, substrate availability, and host–microbe compartmentalization (Mukhopadhya and Louis, 2025). Second, *Escherichia*, *Citrobacter amalonaticus*, PTS, *E. coli* biofilm formation, transporter-related features, and central carbon programs were enriched. This combination fits newer CRC microbiome thinking, in which facultative/pathobiont-associated taxa and functions are interpreted through ecological adaptation, genotoxic potential, biofilm organization, and metabolic niche construction rather than through the presence of one named pathogen (Ecklu-Mensah et al., 2023; Thulasinathan et al., 2025).

The paired stool–serum analysis strengthens this interpretation by connecting microbial ecology with circulating metabolites in the same individuals. The inverse *Roseburia*–succinate relationship is biologically informative, but its FDR value of 0.062 supports an exploratory interpretation. Available dicarboxylate/TCA-axis metabolites were further reviewed: *Roseburia intestinalis* CAG13 showed inverse correlations with succinate (rho = −0.468, nominal *p* = 0.0054, FDR = 0.131) and fumarate (rho = −0.404, nominal *p* = 0.0228, FDR = 0.169), whereas genus-level *Roseburia* showed a weaker association with fumarate (rho = −0.212, nominal *p* = 0.221, FDR = 0.421) and little association with succinic semialdehyde (rho = 0.127, nominal *p* = 0.457, FDR = 0.594). Succinate and related dicarboxylate signals should not be overinterpreted as proof of a single pathway or causal direction; they are better viewed as host-facing evidence that the LST-associated microbial shift has metabolic correlates. Similarly, lower 3-hydroxybutyric acid and altered amino acid and choline/glycerophospholipid signals support systemic metabolic remodeling; however, they do not, by themselves, identify the tissue source of the metabolites or distinguish tumor, host, diet, and microbial contributions.

The CES score should also remain a quantitative summary rather than a diagnostic model. The score shows that prespecified LST-associated features can be integrated into a sample-level measure across stool, serum, and paired multi-omics layers. However, it was not trained as a classifier, was not independently validated for clinical prediction, and should not be used to claim diagnostic performance. Its value is conceptual and analytical: It demonstrates that the LST-associated ecological and metabolic displacement is sufficiently coherent to be summarized within individuals, including through a compact five-anchor representation based on *Roseburia* depletion, succinate elevation, PTS elevation, 3-hydroxybutyric acid depletion, and *Escherichia* enrichment (Li L. et al., 2025).

Several limitations define the boundary of the present claims. The analysis was observational and single-center and could not establish causal relationships among microbial taxa, microbial functions, serum metabolites, and LST development. The cohort did not include a direct ordinary adenoma or serrated lesion comparator, limiting claims about LST specificity. The paired control subset was small; therefore, cross-layer network results should be regarded as supportive evidence of coordination rather than a mechanistic map. Public disease-sequence comparison was limited only to stool microbial features and cannot validate serum metabolic or paired stool–serum findings. Detailed information on diet, smoking, alcohol exposure, and medication history was not systematically collected for covariate adjustment. Finally, microbial feature matching, KEGG pathway interpretation, and metabolite annotation require caution, particularly for CAG-like taxa, proxy functional features, and metabolites with ambiguous biological sources.

Despite these limitations, the study provides a modern, clinically grounded CES framework for LST. It connects an endoscopically important premalignant phenotype with microbial ecology, microbial functional capacity, systemic metabolism, paired cross-layer coordination, and external stool microbial disease-sequence context. The next step is not to present the CES as a completed biomarker but to test it in cohorts that include normal controls, conventional adenomas, serrated lesions, LST subtypes, and CRC cases under harmonized sampling and sequencing conditions. Longitudinal sampling before and after endoscopic resection, mucosal tissue validation, medication and diet metadata, and targeted experimental models will be needed to determine whether the CES is a driver, passenger, or measurable consequence of high-risk colorectal premalignancy.

## Conclusion

LST is associated with a carcinogenic eco-metabolic shift involving protective anaerobe depletion, facultative/pathobiont-associated adaptation, serum metabolic remodeling, and sample-level microbial and metabolic displacement. Public disease-sequence comparison suggests that the matched microbial CES component has a CRC-oriented context, while the LST stool–serum data define the full multi-omics framework. These findings support the CES as a testable model for high-risk premalignant colorectal biology rather than as a causal mechanism or diagnostic test.

## Data Availability

The original metagenomic data were deposited in the NCBI under Bioproject accession number PRJNA1212478: https://www.ncbi.nlm.nih.gov/bioproject/PRJNA1212478.

## Footnotes

1
https://doi.org/10.57745/7IVO3E
